# Risk of ambulance services associated with ambient temperature, fine particulate and its constituents

**DOI:** 10.1038/s41598-021-81197-5

**Published:** 2021-01-18

**Authors:** Yu-Kai Lin, Chia-Pei Cheng, Ho Kim, Yu-Chun Wang

**Affiliations:** 1 Department of Health and Welfare, University of Taipei College of City Management, 101 Zhongcheng Road Sec. 2, Taipei, 111 Taiwan; 2 Department of Environmental Engineering, College of Engineering, Chung Yuan Christian University, 200 Chung-Pei Road, Zhongli, 320 Taiwan; 3 Department of Epidemiology and Biostatistics, School of Public Health, Seoul National University, 103 Daehak-ro, Jongno-gu, Seoul, 03080 Republic of Korea; 4 Research Center for Environmental Changes, Academia Sinica, 128 Academia Road, Section 2, Nankang, Taipei, 11529 Taiwan

**Keywords:** Risk factors, Ecological epidemiology

## Abstract

Short-term adverse health effects of constituents of fine particles with aerodynamic diameters less than or equal to 2.5 μm (PM_2.5_) have been revealed. This study aimed to evaluate the real-time health outcome of ambulance services in association with ambient temperature and mass concentrations of total PM_2.5_ level and constituents in Kaohsiung City, an industrialized city with the worst air quality in Taiwan. Cumulative 6-day (lag0-5) relative risk (RR) and 95% confidence interval (CI) of daily ambulance services records of respiratory distress, coma and unconsciousness, chest pain, headaches/dizziness/vertigo/fainting/syncope, lying at public, and out-of-hospital cardiac arrest (OHCA) in association with ambient temperature and mass concentrations of total PM_2.5_ level and constituents (nitrate, sulfate, organic carbon (OC), and elemental carbon (EC)) from 2006 to 2010 were evaluated using a distributed lag non-linear model with quasi-Poisson function. Ambulance services of chest pain and OHCA were significantly associated with extreme high (30.8 °C) and low (18.2 °C) temperatures, with cumulative 6-day RRs ranging from 1.37 to 1.67 at the reference temperature of 24–25 °C. Daily total PM_2.5_ level had significant effects on ambulance services of lying at public and respiratory distress. After adjusting the cumulative 6-day effects of temperature and total PM_2.5_ level, RRs of ambulance services of lying at public associated with constituents at 90th percentile versus 25th percentile were 1.35 (95% CI: 1.08, 1.68) for sulfate and 1.20 (95% CI: 1.02, 1.41) for EC, while RR was 1.31 (95% CI: 1.09–1.58) for ambulance services of headache/dizziness/vertigo/fainting/syncope in association with OC at 90th percentile versus 25th percentile. Cause-specific ambulance services had various significant association with daily temperature, total PM_2.5_ level, and concentrations of constituents. Elemental carbon may have stronger associations with increased ambulance services than other constituents.

## Introduction

Numerous reports have indicated that risks of morbidities and mortalities are associated with ambient concentration of fine particles with aerodynamic diameters less than or equal to 2.5 μm (PM_2.5_)^1–5^. In a cohort of 4.5 million US veterans, in comparison with a theoretical minimum risk exposure level of 2.4 μg/m^3^^6^, results indicated that the 99% mortality burden of nonaccidental causes associated with ambient PM_2.5_ level is below the US national annual PM_2.5_ standard (12 μg/m^3^)^4^. Researchers identified the source apportionments of PM_2.5_ including industrial emission (or combustion-related source), motor vehicular exhaust, secondary aerosol (secondary nitrate and secondary sulfate), soil dust, natural source, and others (e.g., cooking). Thus, short- and long-term health risks associated with constituents of PM have been comprehensively reviewed^1–3,5,7,8^.

Most studies identified the robust short-term effects of ambient black and organic carbon on mortality from and morbidity of all causes and cardiovascular diseases^1,3,5^. Study conducted in China found that the effects of PM_2.5_ on respiratory diseases were getting stronger after 50 h exposures of it^9^. By contrast, no PM constituent had consistent significant risks under long-term exposure^2,7^. The constituents of PM_2.5_, which vary by study area, source, and season, resulting in various toxicities, are worthy of investigation^3,10–12^.

Previous studies evaluating temperature-health associations often used mortality, outpatients visits, or emergency room visits as health outcome in their studies^8,13,14^. Risks of ambulance dispatch services are rarely evaluated due to a lack of sufficient records. Ambulance call-out data provide new and valuable real-time information that is useful to assess the impact of environmental conditions, such as temperature and air pollution upon human health^15^.

Previous study conducted in Taiwan reported that pollutant constituents were attributable to anthropogenic emissions^12^. A China study observed that emissions from coal burning activities were common dominant sources of OC and EC, they also found that industrial area had higher concentration compared with urban area and coastal area^16^. Compare to other cities in Taiwan, the main sources of air pollution in Kaohsiung City is a combination between urban and industrial areas with a high proportion of factories and petrochemical industry clusters^17^. Even though the air pollution condition in Taiwan is not as severe as Beijing or Dehli, air pollution is still a major issue and has repeatedly induce public concerns in Central and Southern Taiwan since 2010^18^. Therefore, this study aimed to evaluate the risks of ambulance services of respiratory distress, coma and unconsciousness, chest pain, headaches/dizziness/vertigo/fainting/syncope, lying at public, and out-of-hospital cardiac arrest (OHCA) in association with total mass and constituents of fine particulate matters of 2.5 μm in Kaohsiung City, an industrialized city of Taiwan, using records of PM_2.5_, and its constituents (elemental carbon (EC), organic carbon (OC), nitrate, and sulfate), from 2006 to 2010.

## Materials and methods

### Data sources

This study collected the ambulance services data of Kaohsiung City from the Ministry of Health and Welfare from 2006 to 2010. The information and privacy protection of the ambulance services database was described in a previous report^19^. Daily ambulance services records of cases diagnosed with respiratory distress, coma and unconsciousness, chest pain, headaches/dizziness/vertigo/fainting/syncope, lying at public, and OHCA were extracted from non-accidental disease code and analyzed for the association analysis with ambient environmental conditions.

**Figure 1:**
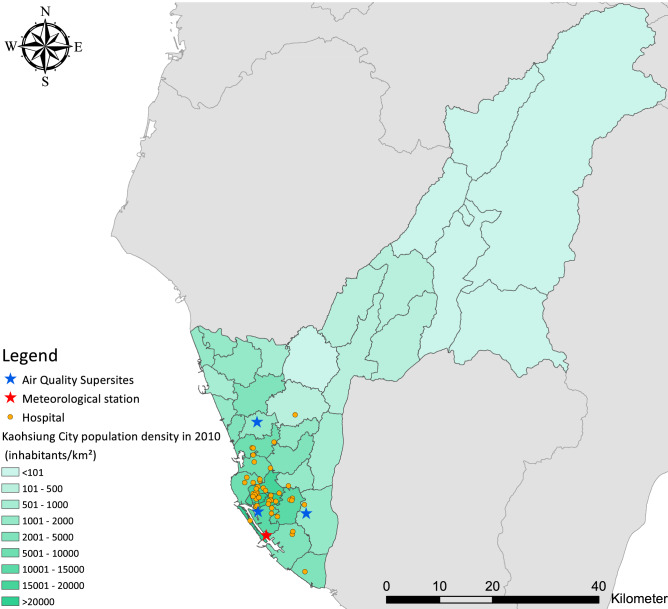
Locations of weather observatories and ambient air quality monitoring stations and district-level population density in 2010 in Kaohsiung City. Generated with ArcGIS Version 10.7 (http://www.esri.com/software/arcgis).

The daily meteorological data, including average air temperature (^o^C), relative humidity (%), wind speed (m/s), were monitored at Kaohsiung surface meteorological observatory (station No. 467440) and collected from the Taiwan Central Weather Bureau. Hourly records of PM_2.5_, and its constituents (elemental carbon (EC), organic carbon (OC), nitrate, and sulfate) from 2006 to 2010 that were measured at three air quality supersites (Fooyin, Qiaotou, and Qianzhen stations; Fig. 1) were collected from the Taiwan Air Quality Monitoring Network**.** The sampling heights at the Fooyin, Qiaotou, and Qianzhen stations supersites are 4.7 m, 14.8 m, 11.1 m^10^. Sunset OC/EC (Sunset Laboratory, USA) helped to measure the continuous PM_2.5_, OC and EC in Kaohsiung City^20^. Studies conducted by Lin et al. (2008) and Kuo et al. (2011) provide detailed information on the monitoring instruments, stations, and quality assurance criteria related to the air quality supersites in Kaohsiung City.

The daily mean temperature and mass concentrations of PM_2.5_ and its constituents (nitrate, sulfate, OC, and EC) were calculated and evaluated in a risk association model.

### Statistical models

#### Non-linear risk association

A distributed lag non-linear model (DLNM) with quasi-Poisson function proposed by Gasparrini et al.^21^ was applied to assess the time-series non-linear exposure–response relationship between the daily mean measurements of temperature, mass concentrations of PM_2.5_, and its constituents (EC, OC, nitrate, and sulfate) and cause-specific number of ambulance services. The model was specified as

$$\mathrm{Log}\left[\mathrm{Y}\right] \sim \mathrm{ BS}\left(\mathrm{T},\mathrm{ lag\, }6\mathrm{\,day}\right)+\mathrm{ BS}\left({\mathrm{PM}}_{2.5}\mathrm{\,or \,constituents},\mathrm{\,lag\, }6\mathrm{\,day}\right)+\mathrm{NS}\left(\mathrm{time},7\mathrm{\,per\,year}\right)+\mathrm{NS}\left(\mathrm{ws},5\right)+\mathrm{ NS}\left(\mathrm{rh},4\right)+\mathrm{ holiday\, effect}+\mathrm{ dow}+\mathrm{PI}$$

where* Y* is the daily cause-specific number of ambulance services, and *T* is the daily mean temperature. We used the B-splines (*BS*) function with two equal knots for average temperature and two knots for lag response to estimate the lag-response risk association. The reference temperature, the temperature with the lowest number for cause-specific ambulance services, was evaluated. Overall 6-day (lag0–5) relative risk (RR) and related 95% confidence interval (CI) of cause-specific ambulance services associated with extreme temperatures (5th and 99th percentiles) were estimated and displayed, controlling for daily mass concentration of PM_2.5_.

Daily mass concentrations of PM_2.5_ and its constituents, set as *BS* function with 2 degrees for measurements and lag effect, were evaluated and included in the model separately. Cumulative 6-day risks of cause-specific ambulance services associated with mass concentration of PM_2.5_ were evaluated and displayed at 90th percentile of measurements, controlling for daily average temperature. The reference concentration was set at Q1 measurements. The cumulative 6-day risks of cause-specific ambulance services associated with mass concentration of PM_2.5_ constituents, controlling for daily average temperature and mass concentration of PM_2.5_, were evaluated and displayed in concentration and percentile scales.

The daily wind speed (ws) and relative humidity (rh) were included in the model and set as the natural spline (*NS*) function with 4 *df*, respectively. Time is included in the model for controlling long-term trend and seasonality^22^. *Dow* is dummy variable for controlling “day of a week effect” in the model^9^. Daily deaths from pneumonia and influenza (PI) were also included in the model.

All analyses in this study were carried out using the *mgcv* and *dlnm* packages in RStudio Version 1.2.1335 (http://www.R-project.org/).

### Ethics approval and consent to participate

All methods were carried out in accordance with relevant guidelines and all protocols were approved by Taiwan National Health Research Institutes (code: EC1050701-E).

## Results

### Descriptive characteristics of ambient environment and ambulance services

In Kaohsiung, Taiwan, the proportions of ambulance services were 3.74% for respiratory distress, 3.04% for coma and unconsciousness, 1.99% for chest pain, 5.12% for headache/dizziness/vertigo/fainting/syncope, 3.33% for lying at public, and 2.04% for OHCA from 2006 to 2010. The daily average case numbers of ambulance services were 4.59 (range: 0–13) for respiratory distress, 3.73 (range: 0–12) for coma and unconsciousness, 2.44 (range: 0–10) for chest pain, 6.28 (range: 0–17) for headache/dizziness/vertigo/fainting/syncope, 2.40 (range: 0–9) for lying at public, and 2.51 (range: 0–11) for OHCA from 2006 to 2010 in Kaohsiung City (Table 1). No seasonal variations in daily numbers of cause-specific ambulance services were observed (Supplementary Fig. S1).

**Table 1:** Descriptive statistics for daily cause-specific ambulance services, mass and constituents concentrations of fine particulate matter (PM_2.5_), and weather measurements from 2006 to 2010.

	Mean	SD	Min	10th	25th	50th	75th	90th	Max	IQR	Missing values (%)
PM_2.5_ total mass (µg/m^3^)	33.3	16.2	6.92	13.1	19.3	31.9	44.6	55.1	120	25.4	0.05
**PM**_**2.5**_** constituents (µg/m**^**3**^**)**											
Nitrate	4.35	3.29	0.34	0.79	1.49	3.82	6.42	8.98	21.54	4.93	0.16
Sulfate	9.37	4.80	1.09	3.30	5.45	8.98	12.59	15.83	32.86	7.14	0.22
Organic carbon	8.00	3.74	1.38	3.76	5.10	7.30	10.38	13.22	27.07	5.28	1.20
Elemental carbon	2.21	0.90	0.43	1.31	1.59	2.04	2.66	3.39	13.15	1.07	1.20
**Meteorological variable**											
Temperature (°C)	25.4	3.80	13.5	19.9	22.7	26.4	28.5	29.6	31.5	5.82	0
Relative humidity (%)	75.0	6.83	45.4	66.8	71.1	74.9	78.8	83.2	99.6	7.71	0
**Emergency ambulance dispatches call help (cases/day)**											
Respiratory distress	4.59	2.33	0.00	2.00	3.00	4.00	6.00	8.00	13.0	3.00	0
Coma and unconsciousness	3.73	2.05	0.00	1.00	2.00	4.00	5.00	7.00	12.0	3.00	0
Chest pain	2.44	1.70	0.00	0.00	1.00	2.00	3.00	5.00	10.0	2.00	0
Headache/dizziness/vertigo/fainting/syncope	6.28	2.61	0.00	3.00	4.00	6.00	8.00	10.0	17.0	4.00	0
Lying at public	2.40	1.58	0.00	1.00	1.00	2.00	3.00	4.00	9.00	2.00	0
Out-of-hospital cardiac arrest	2.51	1.80	0.00	0.00	1.00	2.00	4.00	5.00	11.0	3.00	0

**Figure 2:**
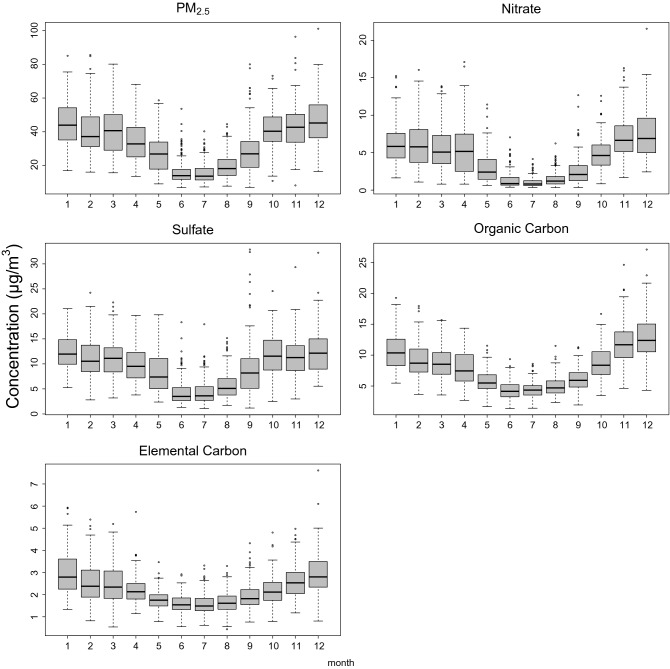
Boxplots for concentrations of mass and constituents of fine particulate matter (PM_2.5_) by month from 2006 to 2010 in Kaohsiung City. Generated with RStudio Version 1.2.1335 (http://www.R-project.org/) using packages ‘mgcv’ and ‘dlnm’ (The box encloses the interquartile range with the lower edge at the first quartile, *q*_*1*_*,* upper edge at the third quartile, *q*_*3*_. A line is drawn through the box at the second quartile (which is the 50th percentile or median). A whisker extends from each end of the box which is the smallest and largest values.

The daily mean temperature was 25.4 °C (range: 13.5–31.5 °C), with mean PM_2.5_ concentration of 33.3 μg/m^3^ (range: 6.92–119 μg/m^3^). The mass concentrations were 4.35 μg/m^3^ (range: 0.34–21.5 μg/m^3^) for nitrate, 9.37 μg/m^3^ (range: 1.09–32.9 μg/m^3^) for sulfate, 8.00 μg/m^3^ (range: 1.38–27.1 μg/m^3^) for OC, and 2.21 μg/m^3^ (range: 0.43–13.2 μg/m^3^) for EC (Table 1). The annual concentrations of PM_2.5_, sulfate, and EC decreased from 2006 to 2010, but the concentrations of nitrate and OC remained stable during the study period (Supplementary Fig. S2). Figure 2 shows the boxplots for average mass concentrations of PM_2.5_, and its constituents by month. The average mass concentrations were lower in summer (June to August), but the proportions of EC and OC of PM_2.5_ were particularly high in summer (Supplementary Fig. S3).

### Relative risk of ambulance services associated with daily ambient temperature and PM_2.5_ concentration

**Figure 3:**
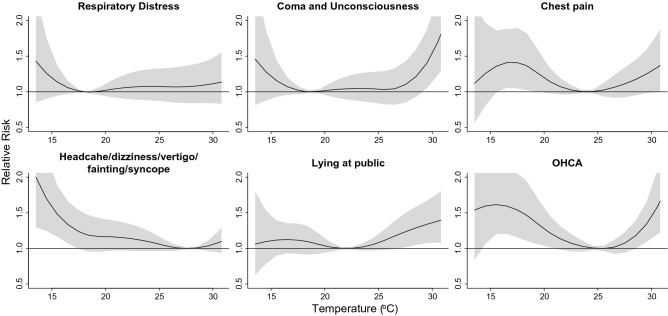
Cumulative 6-day risks of cause-specific ambulance services associated with ambient temperature in Kaohsiung City from 2006 to 2010. Generated with RStudio Version 1.2.1335 (http://www.R-project.org/) using packages ‘mgcv’ and ‘dlnm’.

Figure 3 shows the cumulative 6-day RR of cause-specific ambulance services in association with daily average temperature. The temperatures for the lowest number of ambulance services (reference temperature) were 18 °C for respiratory distress, 19 °C for coma and unconsciousness, 24 °C for chest pain, 28 °C for headache/dizziness/vertigo/fainting/syncope, 22 °C for lying at public, and 25 °C for OHCA. Ambulance services of coma and unconsciousness, chest pain, lying at public, and OHCA were elevated at extremely high temperature; the RR ranged from 1.37 to 1.81 at the 99th percentile of temperature (Table 2). By contrast, increased ambulance services of chest pain, headache/dizziness/vertigo/fainting/syncope, and OHCA were associated with extreme low temperature, with RR ranging from 1.19 to 1.48 at the 5th percentile of temperature (Table 2). Supplementary Fig. S4 shows temperature-related lag effects (lag0-5) on cause-specific ambulance services. The risks of ambulance services associated with heat peaked at lag0-1, but risks of ambulance services peaked at various lag days in cold environments.

**Table 2:** Cumulative 6-day (lag0-5) relative risks (RR) and related 95% confidence intervals (CI) for cause-specific ambulance services associated with daily average temperature at 5th and 99th percentiles and concentration of fine particulate matter (PM_2.5_) at 90th percentile.

	Temperature at 5th percentile (18.2 °C)		Temperature at 99th percentile (30.8 °C)		PM_2.5_ at 90th percentile (55.1 μg/m^3^)	
	RR	95% CI	RR	95% CI	RR	95% CI
Respiratory distress	1.00	0.99–1.00	1.14	0.83–1.56	1.14	0.98–1.32
Coma and unconsciousness	1.00	0.97–1.04	1.81	1.29–2.53	0.99	0.84–1.16
Chest pain	1.38	1.03–1.84	1.37	1.00–1.89	0.95	0.78–1.16
Headache/dizziness/vertigo/fainting/syncope	1.19	0.96–1.48	1.10	0.94–1.29	1.00	0.88–1.13
Lying at public	1.10	0.91–1.32	1.39	1.08–1.81	1.17	1.00–1.37
Out-of-hospital cardiac arrest	1.48	1.11–1.97	1.67	1.23–2.26	1.07	0.88–1.30

**Figure 4:**
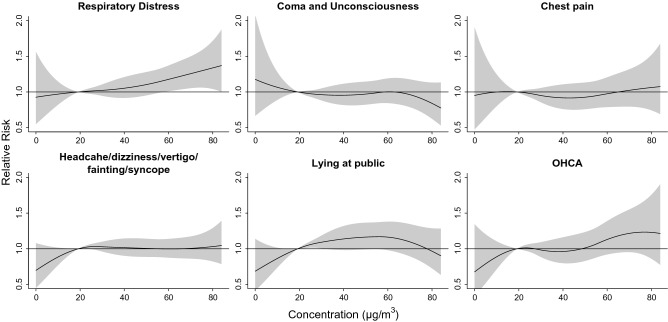
Cumulative 6-day risks of cause-specific ambulance services associated with mass concentrations of fine particulate matter (PM_2.5_) in Kaohsiung City from 2006 to 2010. Generated with RStudio Version 1.2.1335 (http://www.R-project.org/) using packages ‘mgcv’ and ‘dlnm’.

Figure 4 shows the cumulative 6-day RR of cause-specific ambulance services in association with daily PM_2.5_ concentration. This study identified that only ambulance services of respiratory distress, lying at public, and OHCA increased as the PM_2.5_ concentration increased, with RR of 1.07–1.14 at 55.1 μg/m^3^ (90th percentile vs. 25th percentile; Table 2). Risk of ambulance services of lying at public was significantly associated with PM_2.5_ concentration ranging from 20 μg/m^3^ to 60 μg/m^3^, while the risk was significant for ambulance services of respiratory distress as PM_2.5_ concentration exceeded 60 μg/m^3^. Supplementary Fig. S5 shows lag effects (lag0-5) of the PM_2.5_ concentration on cause-specific ambulance services. Risk of ambulance services of respiratory distress, chest pain, and OHCA generally peaked at lag 0–1 in a high PM_2.5_ concentration environment.

### Relative risk of ambulance services associated with daily concentrations of PM_2.5_ constituents

**Figure 5:**
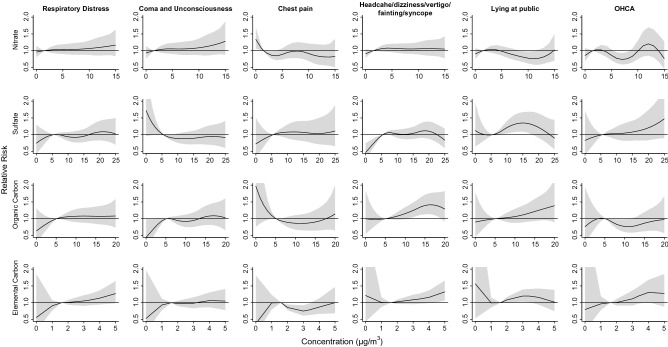
Cumulative 6-day risks of cause-specific ambulance services associated with mass concentrations of PM_2.5_ constituents. Generated with RStudio Version 1.2.1335 (http://www.R-project.org/) using packages ‘mgcv’ and ‘dlnm’.

Figure 5 illustrates the RR of cause-specific ambulance services associated with concentrations of PM_2.5_ constituents. After controlling for cumulative 6-day effects of temperature and PM_2.5_ concentration, the risk associations in percentile scale are displayed in Supplementary Fig. S6. Risks of ambulance services of respiratory distress and coma and unconsciousness increased as the nitrate concentration increased. Ambulance service of lying at public increased as the sulfate concentration was at 90th percentile (15.8 μg/m^3^) with RR of 1.35 (95% CI: 1.08–1.68). The risk of ambulance services of headache/dizziness/vertigo/fainting/syncope was significantly associated with OC concentration at 90th percentile (3.39 μg/m^3^) with RR of 1.31 (95% CI: 1.09–1.58). Elevated EC concentration increased the risks for respiratory distress, headache/dizziness/vertigo/fainting/syncope, lying at public, and OHCA with RRs of 1.08 (95% CI: 0.92–1.26), 1.11 (95% CI: 0.97–1.27) , 1.20 (95% CI: 1.02–1.41), and 1.20 (95% CI: 0.97–1.48) at 90th percentile (13.2 μg/m^3^), respectively.

## Discussion

This study comprehensively evaluated the risk associations between cause-specific ambulance services, extreme temperatures, and mass concentrations of PM_2.5_ and its constituents. The significant cold effects on chest pain and headache/dizziness/vertigo/fainting/syncope and heat effects on coma and unconsciousness and lying at public were observed, while the risk of ambulance services of OHCA was elevated in both extreme heat and cold environments. Ambulance services of respiratory distress, lying at public, and OHCA increased as the PM_2.5_ concentration increased, and the risk was significant at the PM_2.5_ concentration of 20–60 60 μg/m^3^ for ambulance services of lying at public and higher than 60 μg/m^3^ for respiratory distress. After controlling for effects of daily average temperature and PM_2.5_ concentration, this study still identified the significant effects of sulfate and EC on ambulance services of lying at public and OC on headache/dizziness/vertigo/fainting/syncope as the concentrations of PM_2.5_ constituents were at 90th percentile.

Limited studies assessed associations between ambulance calls and ambient environment^9,13,19,22–27^. Studies in Emilia-Romagna in Italy^23^, Brisbane in Australia^26^, Taiwan^19^, and Huainan and Luoyang in China^22,24^, have indicated the numbers of ambulance calls associated with extreme heat; the risks generally increase as the daily temperature exceeds 27 °C^19,23,26^. However, no consistent finding for cold threshold was identified^19,28^. Kaohsiung City has a tropical climate (daily temperature ranging from 13.5 °C to 31.5 °C), but it is cooler than cities located near the equator, e.g., Singapore and Manila. Except for ambulance service of OHCA, we found that the significant risks associated with temperature were only identified in environments with extreme temperatures (< 5th and > 90th percentiles; Fig. 3).

Fine particulate matter (PM_2.5_) are characterized with a small diameter (< 2.5 µm) that can carry various toxic substances and reach the end of the respiratory tract with airflow, accumulate by diffusion, and damage other parts of the body through air exchange in the lungs^29^. A China review reported that organic aerosols account for 20%–45% of PM_2.5_ at sites across China with seasonal and spatial variation^11^. However, as the Taiwan Environmental Protection Administration has declared Kaohsiung City with the worst air quality in Taiwan, particulate sulfate was identified as a primary variable to explain the PM_2.5_ concentration for this metropolitan^10^. This study observed that the proportions of PM_2.5_ constituents were 12.1% for nitrate, 28.2% for sulfate, 25.5% for OC, and 7.56% for EC (Supplementary Fig. S3).

The European Study of Cohorts for Air Pollution Effects (ESCAPE) reported that the hazard ratio of PM_2.5_ should be 1.14 (95% CI: 1.04–1.26) per 10 μg/m^3^ for all-cause mortality^2^. A Korean study reported that the OHCA risk increased for 1.30% after 1 to 2 days exposure to PM_2.5_ by an elevation of 10 μg/m^3^^30^. Our previous study^19^ identified that ambulance events are associated with high concentrations of PM_2.5_ (about 90 μg/m^3^) and significantly elevate the ambulance care for respiratory distress and OHCA. Previous research evaluated the health risks associated with components of PM_2.5_ in recent years^3,5,7,8,14,31–33^. Most studies identified significant short-term effects of PM_2.5_ and its constituents at lag0-2^8,14,33^. Particulate matter significantly increased emergency ambulance dispatched at lag0-1 day in Japan^34^, at lag 0 in Chengdu, China^35^, and Sydney, Australia^36^.

A Beijing study reported combustion-related PM_2.5_ constituents, such as secondary nitrate and sulfate that accounted for 45.9% PM_2.5_ mass concentration, had significant impacts on supraventricular premature beats and atrial tachycardia^31^. Meanwhile, two Shanghai studies reported that the risk of ischemic stroke hospitalization is significantly associated with previous day EC and heavy metal (Cr, Fe, Cu, Zn, As, Se, and Pb) concentrations^33^; moreover, the mortality risks of cardiovascular diseases associated with previous 2-day exposure of OC, sulfate, ammonia, potassium, and heavy metals (Cu, As, and Pb) with RRs ranging from 1.02 to 1.03 per interquartile increase^14^. After adjusting the total PM concentration, hospital admissions of cardiovascular diseases were significantly associated with EC and sulfate of PM_2.5_ at lag 0, and those of respiratory diseases were associated with sulfate at lag 0 and OC and EC at lag 1^8^. On the basis of the findings of the present study and previous reports, the risks of total PM_2.5_ and its constituents could be observed at lag0-2.

A plausible mechanism may explain the short-term association between risk of cardiorespiratory diseases and components of PM_2.5_. One researcher found that elevated blood pressure is associated with ambient increased concentrations of OC, EC, nitrate, and ammonium in patients comorbid with chronic obstructive pulmonary disease^37^. In addition, the linkages between elevations of airway (fractional exhaled nitric oxide)^32^ and circulating inflammatory biomarkers (interleukin-8, tumor necrosis factor-α, and monocyte chemoattractant protein-1)^38^ and increased ambient concentrations of sulfate and potassium at lag 0–2 were identified and reported.

No significant association was reported in a long-term exposure-risk assessment for PM_2.5_ constituents (Cu, Fe, K, Ni, S, Si, V, and Zn) and mortality from cardiovascular diseases in a study involving 19 European cohorts (ESCAPE and TRANSPHORM projects)^2,7^. However, increased risks of dementia, Alzheimer’s disease, autism spectrum disorder, and Parkinson’s disease were significantly associated with long-term PM_2.5_ exposure with RR ranging from 1.16 to 3.26^39^. Migraines are a significant risk factor for Alzheimer’s disease and all‐cause dementia^40^. After adjusting the effects of daily temperature and PM_2.5_, this study identified that increased ambulance service of headache/dizziness/vertigo/fainting/syncope was significantly associated with concentrations of OC and EC. Future studies may discuss detailed biological mechanisms between PM_2.5_ constituents and neurological disorders.

The present study holds several strengths. Confounders, such as the holiday effect, day of the week, long-term trend, and risk associated with infectious pneumonia and influenza, and effects of daily temperature and total PM_2.5_ level were considered in the data analysis models. Risk of cause-specific ambulance services associated with daily concentrations of PM_2.5_ components was evaluated using continuous hourly monitored data. To date, the risk of ambulance service associated with concentrations of PM_2.5_ components requires further study. The findings from this study can be the ground information to minimize the response time of allocation ambulance services, by knowing which variables should be observed and forecasted in the future^41^. In addition, the individuals can adapt their behavior to enhance health and resilience against the negative impacts from temperature and concentrations of mass and constituents of PM_2.5_.

This study also had several limitations. First, our work is an ecological study. Risk was not estimated with individual-based data. Given the lack of information on personal disease history, medicine usage, behaviors of drinking and exercise, and accessibility of medical services, we did not evaluate modifying effects (e.g., socio-economic status) on risk associated with the ambulance events. Cases’ diagnoses in the medical records of ambulance dispatches were based on observations made by medical personnel, and no ICD codes were provided. In addition, this study did not exclude the complicated conditions of ambulance services, including delayed arrival time, restricted service time, and a location hard to reach. Moreover, this study did not collect ions and heavy metal data of gases and aerosols during the study period, so these effects were not evaluated.

Understanding the short-term risk association between ambulance services and ambient environment is a critical concern for organizers of outdoor activities, especially in extreme temperatures and high air pollution events. The present study provides scientific evidence that, other than mortality from and morbidity of cardiorespiratory diseases^3,5^, cause-specific ambulance services, even for general health symptoms like headache/dizziness/vertigo/fainting/syncope, are associated with concentrations of PM_2.5_ constituents after adjusting the effects of daily temperature and total PM_2.5_ level. This study agreed with a review report^3^, who reported that EC has stronger association with health risks that means elevation of ambulance service in the present study. We also agree that constituents and its proportion of PM_2.5_ vary with the study area, thus, health risk assessment for various health outcomes and area are recommended^1,2^.

## Conclusions

This study evaluated how ambulance services, including call helps of respiratory distress, coma and unconsciousness, chest pain, headache/dizziness/vertigo/fainting/syncope, lying in public, and OHCA, were associated with daily temperature, total PM_2.5_ level, and concentrations of constituents in the city with worst air quality, Kaohsiung City, in Taiwan. The temperatures with the lowest ambulance services ranged from 18 to 28 °C for various cause-specific ambulance services. Increased call helps of chest pain and OHCA were associated with both extreme high and low temperatures. In addition, risk of ambulance services of lying at public significantly associated with PM_2.5_ concentration ranged from 20 to 60 μg/m^3^, and the risk of ambulance services of respiratory distress was significant as the PM_2.5_ concentration exceeded 60 μg/m^3^. After adjusting the cumulative 6-day effects of temperature and total PM_2.5_ level, this study observed that ambulance services of headache/dizziness/vertigo/fainting/syncope significantly increased with increased daily OC and EC concentrations. Meanwhile, the risk of ambulance services of lying at public was significantly associated with daily sulfate and EC concentrations. This study provides health risk in association with PM_2.5_ constituents in an industrialized city located in Southeast Asia. Health effects from exposure to hot and humid climate and PM_2.5_ with high toxicity potential were revealed in this study of a simulated scenario for Western industrialized cities in the future.

## Supplementary Information

Supplementary Legends.

Supplementary Figure S1.

Supplementary Figure S2.

Supplementary Figure S3.

Supplementary Figure S4.

Supplementary Figure S5.

Supplementary Figure S6.

## Data Availability

Data not available due to [ethical/legal/commercial] restrictions.
