# Supplementary material for: Risk of ambulance services associated with ambient temperature, fine particulate and its constituents
Source: Sci Rep. 2021 Jan 18;11:1651. doi: 10.1038/s41598-021-81197-5 (PMC7813819; doi:10.1038/s41598-021-81197-5)
Supplement: Supplementary file 1 — Supplementary Legends. [file 41598_2021_81197_MOESM1_ESM.docx]

**Supplementary Figure legends**

Figure S1. Boxplots for cause-specific ambulance services by month from 2006 to 2010 in Kaohsiung City. Generated with RStudio Version 1.2.1335 (<http://www.R-project.org/>) using packages ‘mgcv’ and ‘dlnm’.

Figure S2. Boxplots for concentrations of mass and constituents of particulate matter (PM_10_) and fine particulate matter (PM_2.5_) by year from 2006 to 2010 in Kaohsiung City. Generated with RStudio Version 1.2.1335 (<http://www.R-project.org/>) using packages ‘mgcv’ and ‘dlnm’.

Figure S3. Concentrations and percentages for constituents of fine particulate matter (PM_2.5_) by month from 2006 to 2010 in Kaohsiung City. Generated with RStudio Version 1.2.1335 (<http://www.R-project.org/>) using packages ‘mgcv’ and ‘dlnm’.

Figure S4. Lag-response associations (3D plots) between ambient temperature and cause-specific ambulance services. Generated with RStudio Version 1.2.1335 (<http://www.R-project.org/>) using packages ‘mgcv’ and ‘dlnm’.

Figure S5. Lag-response associations (3D plots) between mass concentrations of fine particulate matter (PM_2.5_) and cause-specific ambulance services. Generated with RStudio Version 1.2.1335 (<http://www.R-project.org/>) using packages ‘mgcv’ and ‘dlnm’.

Figure S6. Cumulative 6-day risks of cause-specific ambulance services associated with concentrations of PM_2.5_ constituents in percentiles scale. Generated with RStudio Version 1.2.1335 (<http://www.R-project.org/>) using packages ‘mgcv’ and ‘dlnm’.
