# Supplementary figures and images for: Risk of ambulance services associated with ambient temperature, fine particulate and its constituents
Source: Sci Rep. 2021 Jan 18;11:1651. doi: 10.1038/s41598-021-81197-5 (PMC7813819; doi:10.1038/s41598-021-81197-5)

PM<sub>2.5</sub>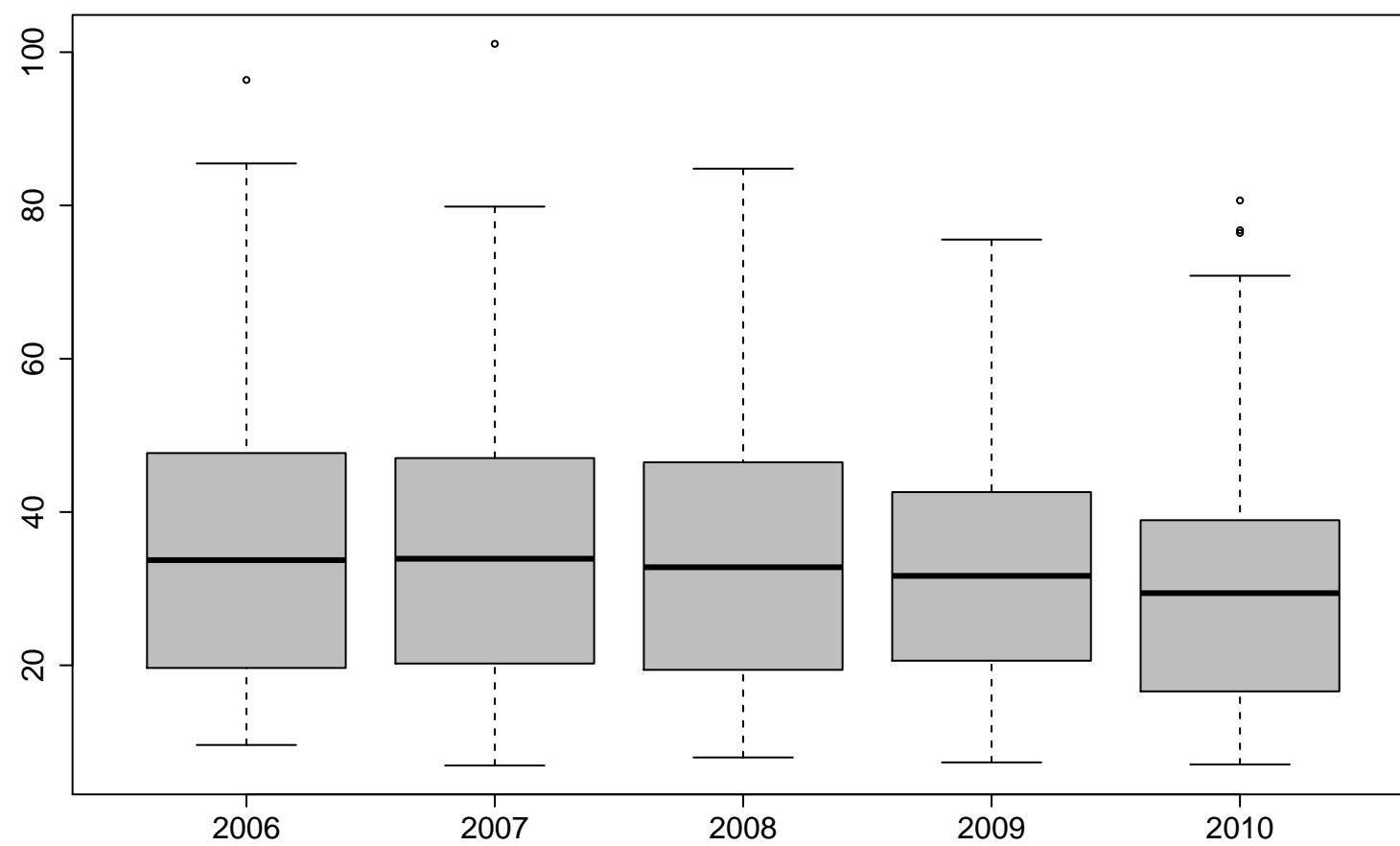

Nitrate

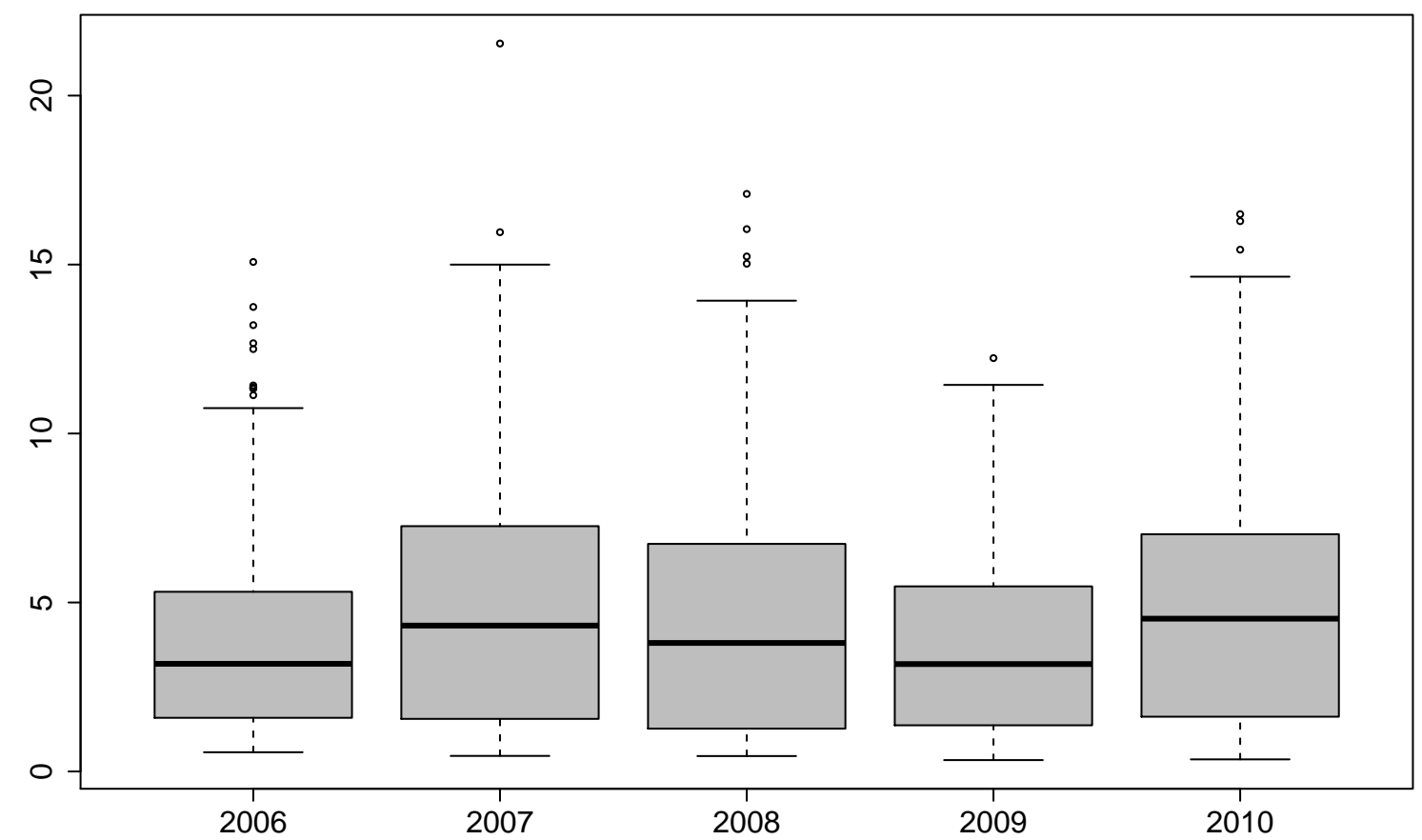

Sulfate

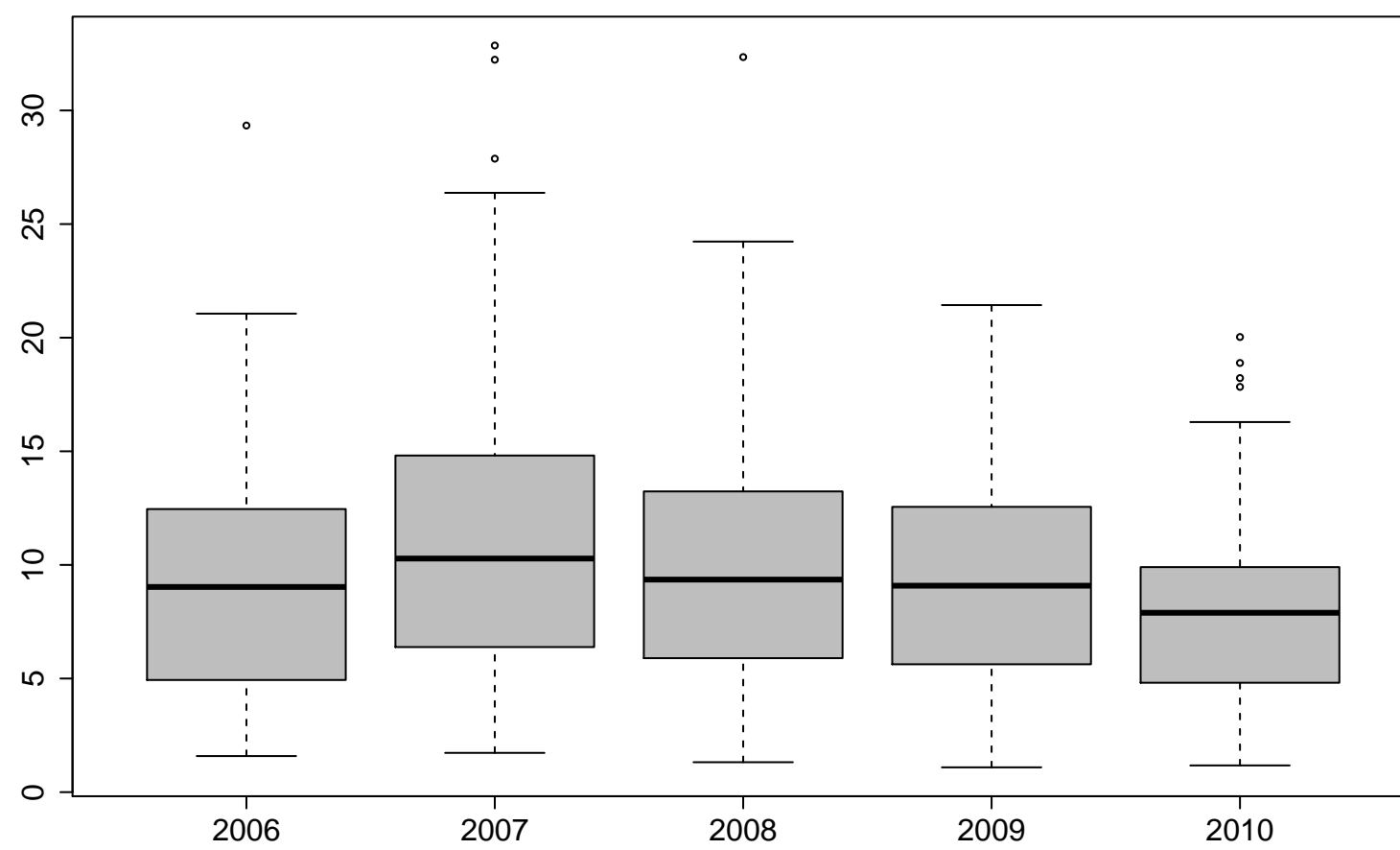

Organic Carbon

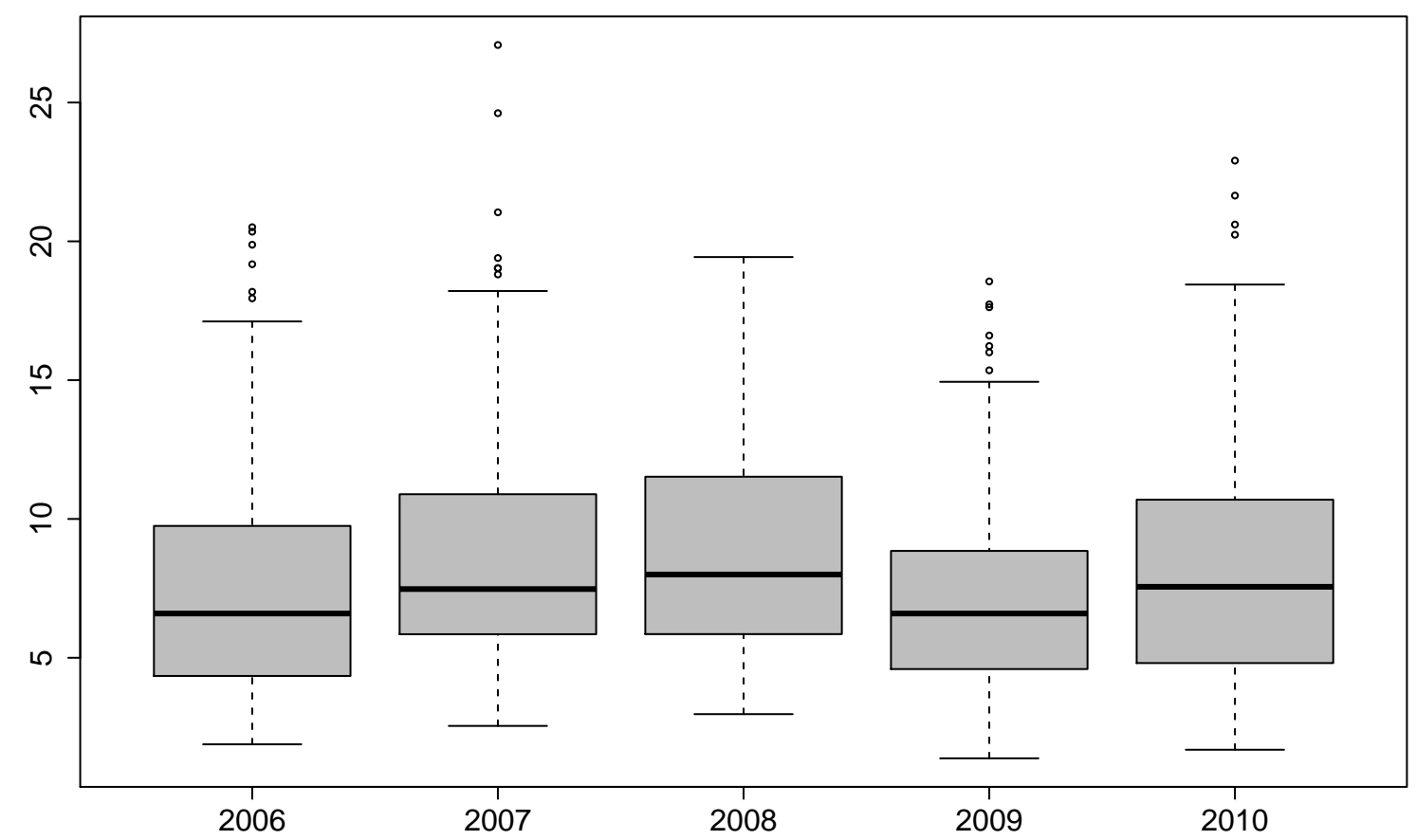

Elemental Carbon

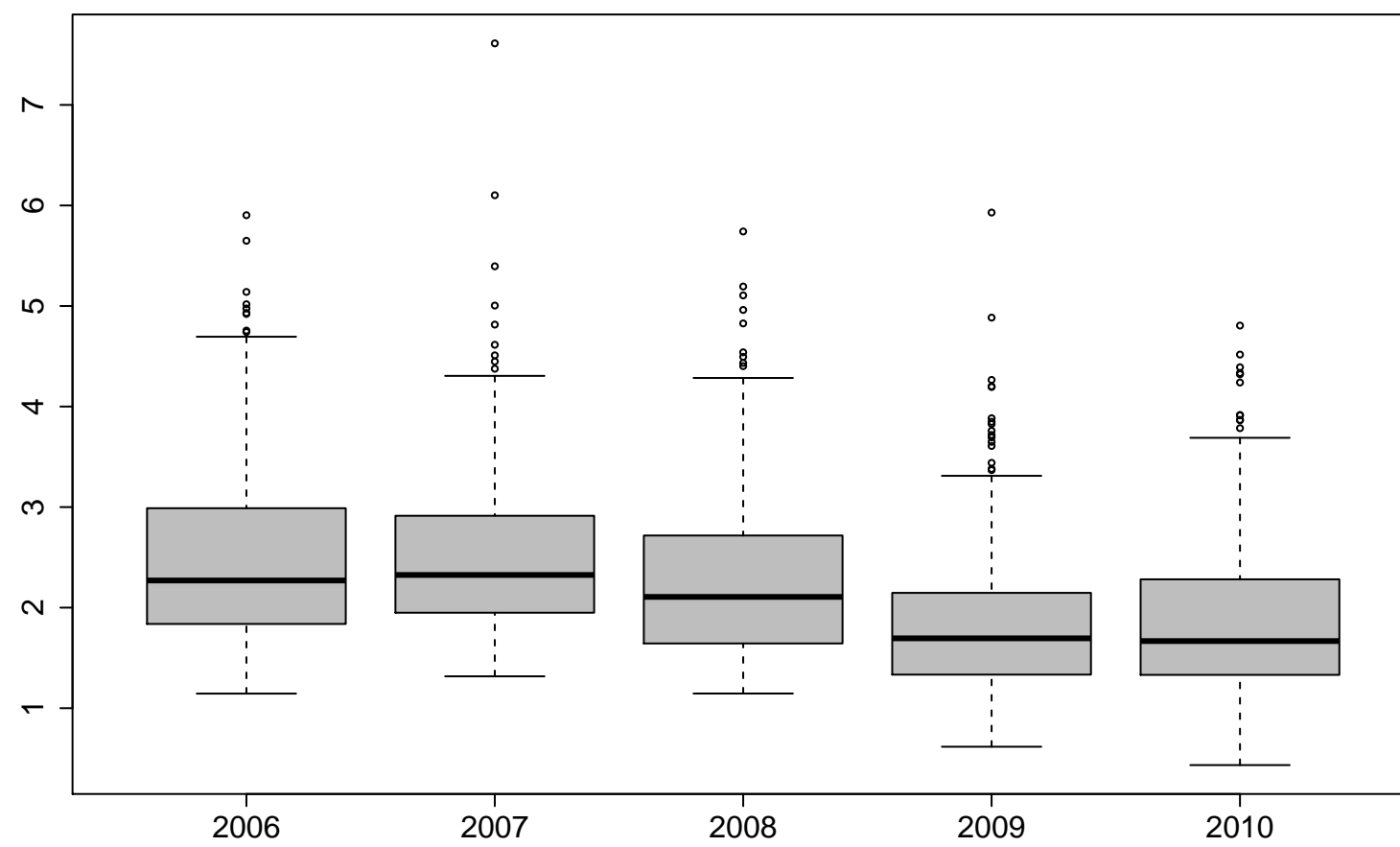

year

Supplement: Supplementary file 3 — Supplementary Figure S2. [file 41598_2021_81197_MOESM3_ESM.pdf]

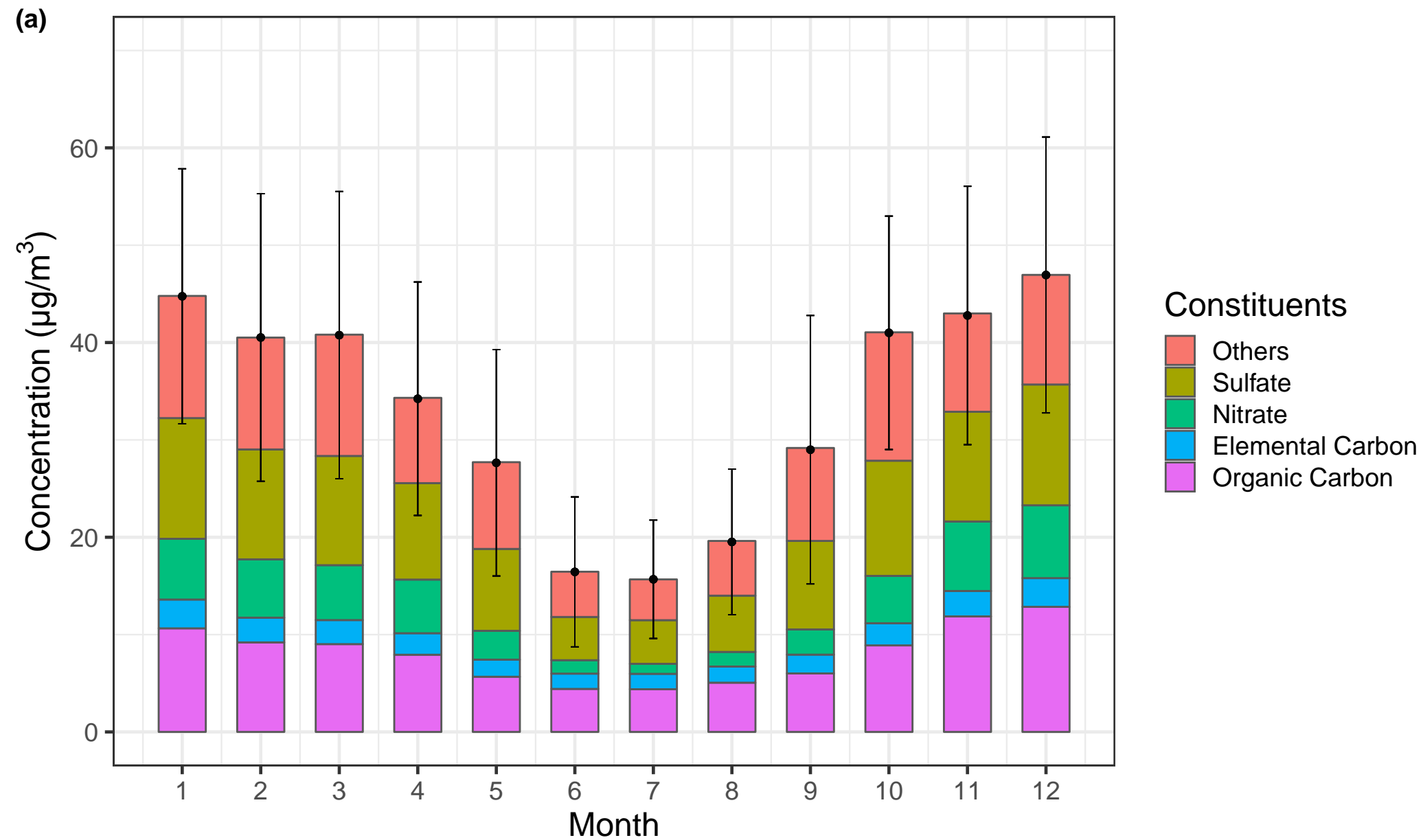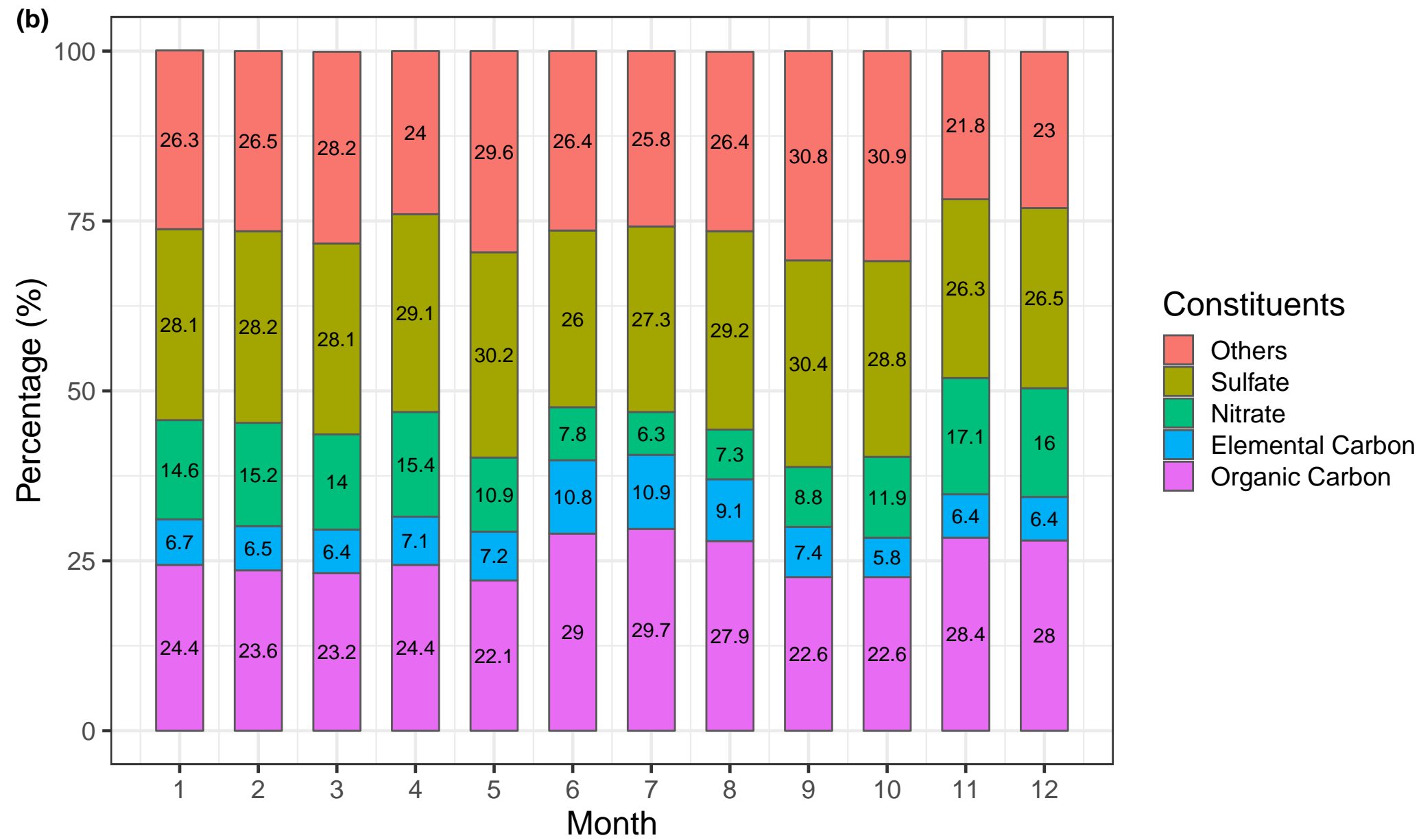

Supplement: Supplementary file 4 — Supplementary Figure S3. [file 41598_2021_81197_MOESM4_ESM.pdf]

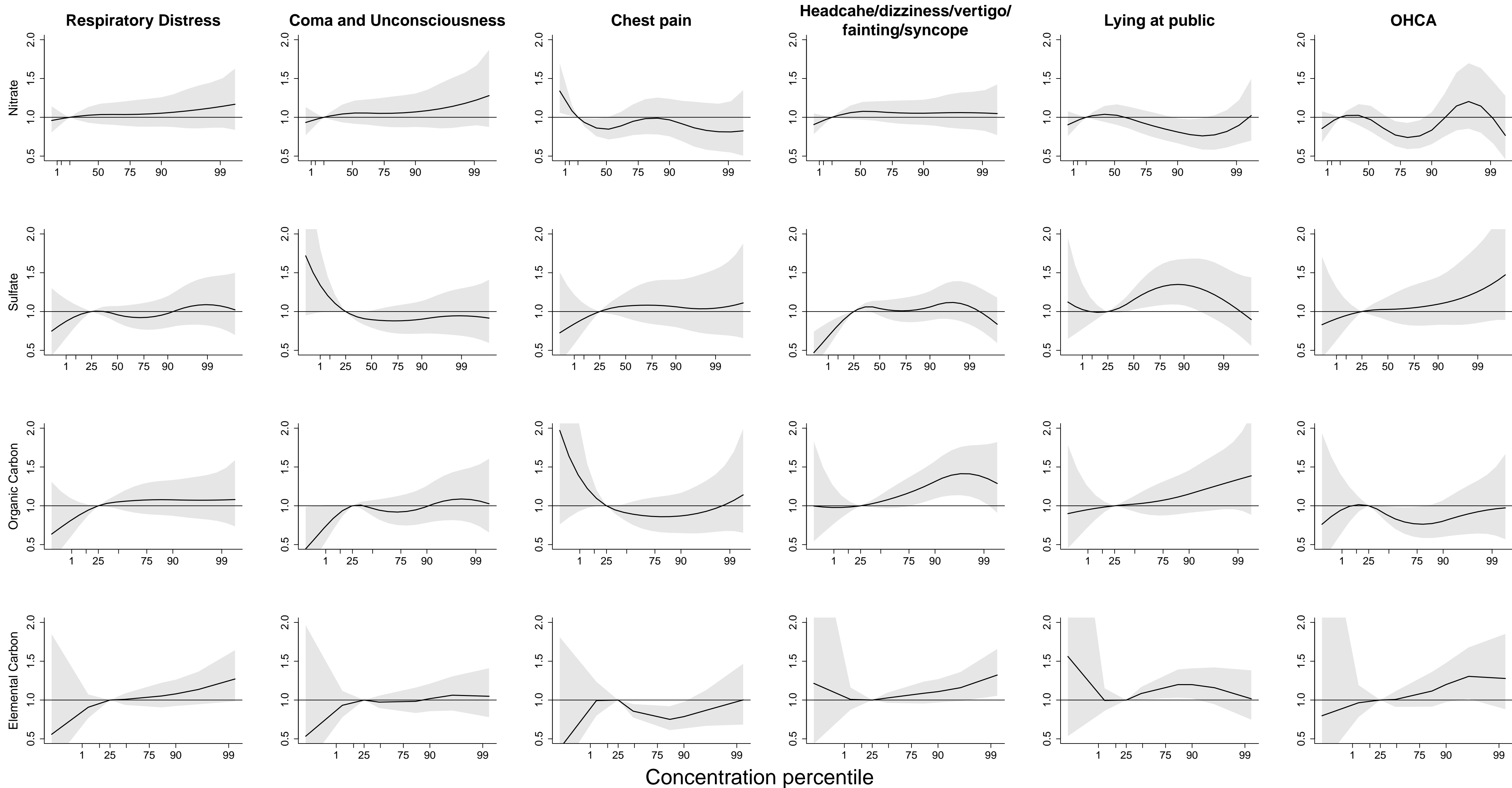

Supplement: Supplementary file 7 — Supplementary Figure S6. [file 41598_2021_81197_MOESM7_ESM.pdf]
