# Supplementary material for: Risk of ambulance services associated with ambient temperature, fine particulate and its constituents
Source: Sci Rep. 2021 Jan 18;11:1651. doi: 10.1038/s41598-021-81197-5 (PMC7813819; doi:10.1038/s41598-021-81197-5)
Supplement: Supplementary file 5 — Supplementary Figure S4. [file 41598_2021_81197_MOESM5_ESM.pdf]

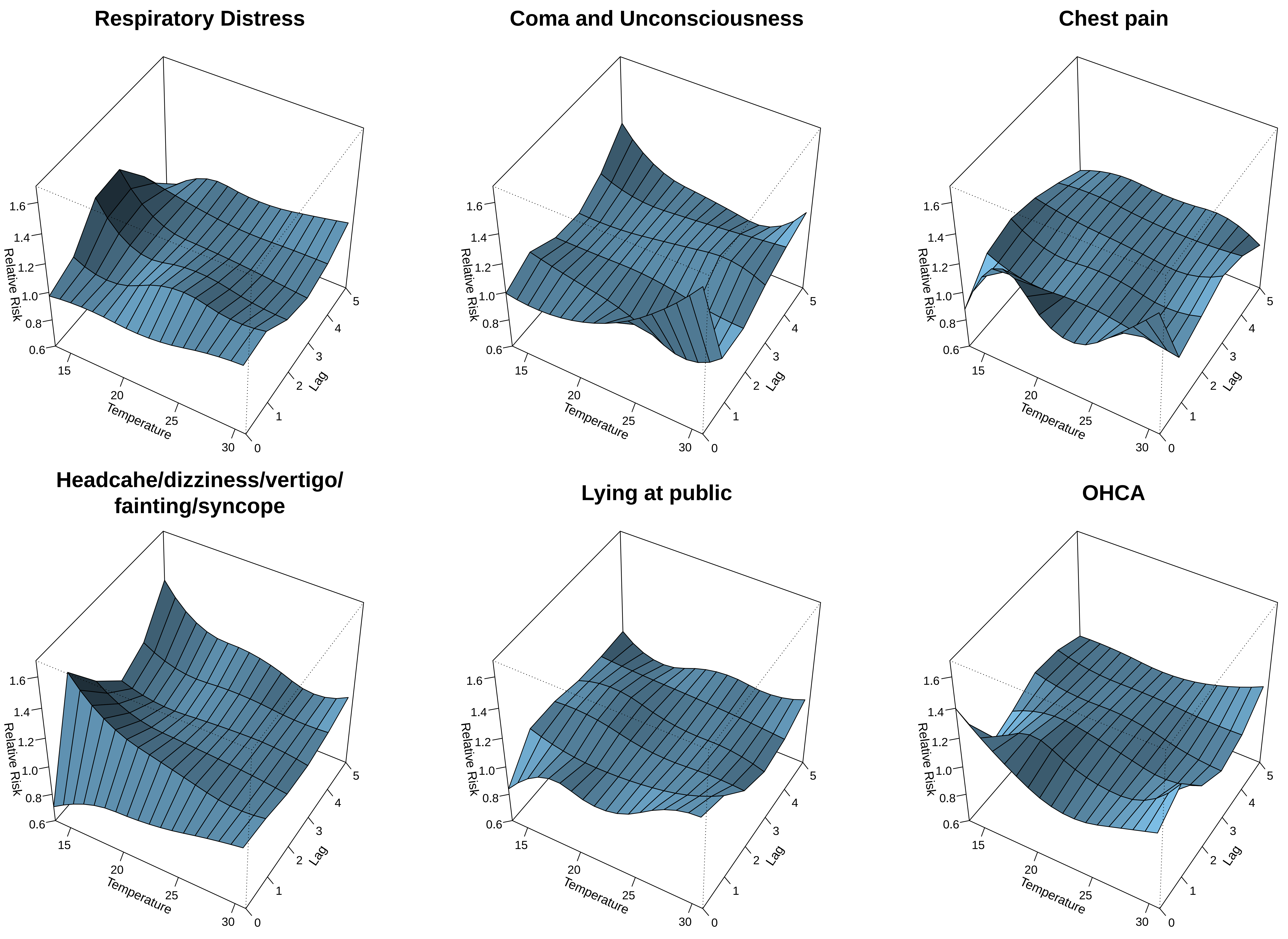
Respiratory Distress
Coma and Unconsciousness
Chest pain
Headcahe/dizziness/vertigo/
fainting/syncope
Lying at public
OHCA
Relative Risk
0.6
0.8
1.0
1.2
1.4
1.6
Temperature
15
20
25
30
Lag
0
1
2
3
4
5
